# Frontotemporal phase lag index correlates with seizure severity in patients with temporal lobe epilepsy

**DOI:** 10.3389/fneur.2022.855842

**Published:** 2022-12-01

**Authors:** Lingyan Mao, Gaoxing Zheng, Yang Cai, Wenyi Luo, Qianqian Zhang, Weifeng Peng, Jing Ding, Xin Wang

**Affiliations:** ^1^Department of Neurology, Zhongshan Hospital, Fudan University, Shanghai, China; ^2^CAS Center for Excellence in Brain Science and Intelligence Technology, Shanghai, China; ^3^Department of the State Key Laboratory of Medical Neurobiology, The Institutes of Brain Science and the Collaborative Innovation Center for Brain Science, Fudan University, Shanghai, China

**Keywords:** temporal lobe epilepsy, phase lag index, frontotemporal network, graph theory, LORETA

## Abstract

**Objectives:**

To find the brain network indicators correlated with the seizure severity in temporal lobe epilepsy (TLE) by graph theory analysis.

**Methods:**

We enrolled 151 patients with TLE and 36 age- and sex-matched controls with video-EEG monitoring. The 90-s interictal EEG data were acquired. We adopted a network analyzing pipeline based on graph theory to quantify and localize their functional networks, including weighted classical network, minimum spanning tree, community structure, and LORETA. The seizure severities were evaluated using the seizure frequency, drug-resistant epilepsy (DRE), and VA-2 scores.

**Results:**

Our network analysis pipeline showed ipsilateral frontotemporal activation in patients with TLE. The frontotemporal phase lag index (*PLI*) values increased in the theta band (4–7 Hz), which were elevated in patients with higher seizure severities (*P* < 0.05). Multivariate linear regression analysis showed that the VA-2 scores were independently correlated with frontotemporal *PLI* values in the theta band (*β* = 0.259, *P* = 0.001) and age of onset (*β* = −0.215, *P* = 0.007).

**Significance:**

This study illustrated that the frontotemporal *PLI* in the theta band independently correlated with seizure severity in patients with TLE. Our network analysis provided an accessible approach to guide the treatment strategy in routine clinical practice.

## Introduction

Epilepsy is one of the most common neurological diseases, affecting ~50 million people worldwide (1). In total, ~30% of patients with epilepsy are drug-resistant and suffer from seizure recurrence (2). Increased intractable rates leading to premature mortality, the global burden of disease, and social stigma have been public health concerns (1). Temporal lobe epilepsy (TLE) accounts for the largest proportion of drug-resistant epilepsy (DRE) (3). The mechanisms of DRE were proposed as alterations in the antiseizure medication (ASM) targets with the loss of receptor sensitivity and overexpression of multidrug resistance (MDR) proteins (MDR1, MDR1/P-glycoprotein, etc.) that affect drug transport (2). In addition, abnormal neural circuits, such as hippocampal–diencephalic–cingulate paths (4), extratemporal networks (5), and dynamic network evolution (6), were suggested to be relevant to seizure severity in TLE. Clinically recognizing potential severities and deciding on treatment strategies for patients with TLE remains a challenge.

Temporal lobe epilepsy is characterized as a neural network disorder that propagates beyond the regions that are anatomically connected (7, 8). The underlying basis of the neural network in TLE involves structural and functional alterations, including neurogenesis and functional plasticity (9). It has been reported that extratemporal networks, such as the orbitofrontal, insular, medial frontal, bifrontal, and parietal regions, were involved in some patients with TLE (7, 10). Indeed, wider spreading networks were spanning more interconnected regions in TLE patients with drug-resistance and focal to bilateral tonic–clonic seizures (11). Furthermore, rapid spread in the lateral temporal cortex was detected in TLE patients with recurrent seizures (12). Several studies have illustrated the anatomical and electrophysiological connections with the extemporal regions in patients with TLE. A typical pathway from the hippocampus to the prefrontal cortex, which is called the hippocampus–prefrontal pathway, has been uniquely reported as a monosynaptic and unidirectional projection mediated by glutamate (13). The lateral temporal, frontal, and occipital neocortexes develop from the same origin of cortical gradients of laminar elaboration and organize comparable neurogenetic time courses. They are connected *via* various fibers, such as the arcuate fasciculus (AF), uncinate fasciculus (UF), cingulate, and longitudinal fasciculus (14). Thus, an accessible tool to distinguish the evolution of the extratemporal network may help differentiate outcomes in patients with TLE.

Functional magnetic resonance imaging (fMRI) and electroencephalography (EEG) were utilized to map the functional epileptic network (15). Using fMRI, a seizure extratemporal propagation network of TLE was identified, including the bilateral thalamus, insula, midcingulate, and precuneus (16). However, MRI measures the blood–oxygen signal and indirectly characterizes electrophysiological functions, regardless of its mental contraindications and expense. In contrast, EEG directly reflects neural activity (15). Stereoelectroencephalography (SEEG) is the gold standard for marking epileptogenic and propagation networks (8) but is limited to invasion, spatial undersampling, and heterogeneous topological signatures for individual electrode placement (17). Scalp EEG offers a standard acquisition and non-invasive scheme for determining the network. However, in terms of its anatomical disadvantages (15), a more precise arithmetic tool based on scalp EEG is required to overcome the challenge of mapping the seizure spread and epilepsy characteristics in TLE.

Graph theory provides a topology of the underlying neural architecture (18). Recently, small-worldness, minimum spanning tree, and community structure are the popular network methods to help clinicians better understand how seizures originate and propagate. Based on EEG, the alternations of local segregation (characterized by clustering coefficient, *CC*) and global integration (characterized by shortest path length, *PL*) were reported in the epileptic network study (19, 20). Compared to other measures (i.e., magnitude squared coherence, synchronization likelihood, phase locking value, etc.), *PLI* was proved to be the least affected functional connectivity (FC) metric by spurious influences in a simulated volume conduction environment (21). Phase lag index (*PLI*), indicating brain synchronization, was decreased with seizure reduction in focal DRE (22). A minimum spanning tree (MST) was introduced into the brain network field to avoid the threshold choice during the network construction (23–25). The clinical application of MST is still lacking because the method is not as popular as the small-world network. In 2016, van Diessen et al. found that MST features are more sensitive to identifying the interictal network alterations than classical weighted network analysis at an early stage of focal epilepsy (26). Thus, the MST was adopted to detect the alternations of the patients with TLE in this study. Community structure quantifies the extent of brain functional network partition and was recently introduced to help classify epileptic EEG events (27). However, the community construction in EEG network analysis always lacks its physiological meaning because of the bad EEG spatial resolution. By using source location techniques, the community analysis may reveal the hidden network mechanisms of the seizure propagation. Distributed EEG source localization using standardized low-resolution brain electromagnetic tomography analysis (sLORETA) is a relatively new method to yield three-dimensional (3D) images of electrical neuronal activity (28). Despite the robustness of indicators that was demonstrated in this approach, it still lacked consistent results and clinical applications. This study aimed to find the brain network indicators based on EEG and provide an accessible approach to indicate seizure severity in patients with TLE and guide the treatment strategy for routine clinical practice.

## Materials and methods

### Participants

A total of 151 patients with TLE, diagnosed and classified according to 2001 (29) and 2017 (30) Classification and Terminology of the International League against Epilepsy (ILAE), were enrolled in the Department of Neurology, Zhongshan Hospital, Fudan University, from April 2018 to December 2019. The inclusion criteria were as follows: (1) patients aged from 14 to 80 years; (2) performed long-term video-EEG (VEEG) monitoring longer than 16 h; (3) no other disease except epilepsy; and (4) no extratemporal lesion on routine cerebral MR.

We enrolled 36 age- and sex-matched controls who underwent VEEG for non-specific symptoms such as dizziness and headache. The control group was excluded if the patients (1) had abnormal VEEG results or routine MRI; (2) had a central nervous system mental illness or other systemic diseases; and (3) had a family history of epilepsy and mental illness.

This study has been approved by the ethics committee. All the participants provided ethical and informed consent.

### Clinical evaluation of patients with TLE

Clinical data were collected at the time of recruitment, including the age of onset, course of the disease, family history, history of febrile convulsions, seizure symptoms (including aura, seizure performance, post-seizure status, duration), ASM schedules, and history of status epilepticus. A routine cerebral MRI was performed for etiology analysis.

Interictal severity was evaluated as seizure frequency, DRE (31), and the clinical scale: VA-2 Veterans administration rating scale for seizure type and frequency (VA-2) (32). VA-2 scale assessed the seizure severity based on the seizure frequency, combined with the seizure manifestations, and was more suitable for assessing interictal severity compared to other scales such as National Hospital Seizure Severity Scale (NHS3) and Liverpool Seizure Severity Scale Items (LSSS).

### EEG acquisition and preprocessing

All participants stayed in a room with attenuated sound and dim light, isolated from electronic devices such as mobile phones, computers, chargers, and continuous video monitoring for more than 16 h. EEG signals were recorded on 25 electrodes (Fp1, Fp2, F9, F10, T9, T10, P9, P10, F7, F8, T7, T8, P7, P8, F3, F4, C3, C4, P3, P4, O1, O2, Fz, Cz, and Pz) positioned according to the IFCN (33) (Supplementary Figure S1), using a 64-channel digital EEG recording system (NIHON KONHDEN, JAPAN). The sampling rate was set at 500 Hz. All the skin/electrode impedances were kept below 5KΩ. At least two EEG specialists interpreted the EEG results.

The 90 s interictal-resting EEG data without noticeable artifacts were collected under the eyes-closing state in patients with TLE and controls. Data with numerous artifacts were eliminated. In patients with epilepsy, the resting EEG was more than half an hour away from a seizure. The EEG data were analyzed using MATLAB R2018a software (MathWorks, Natick, MA) and reformatted into average references to minimize the confounding effects of specific brain activity and achieve reference elimination. A finite impulse response (FIR) filter was used as a band-pass filter for the EEG signal from 0.1 to 45 Hz. Artifacts were removed from each individual blindly by an experienced engineering doctor, Gaoxing Zheng, as far as possible. Independent component analysis (ICA) was performed using the Infomax ICA algorithm in EEGLAB to remove artifacts by identifying and extracting visible artifacts (eye movement, heart activity, and scalp muscle contraction). There was no patient excluded because of the artifacts affecting EEG processing in this study. Band-pass filtering was applied to the following standard frequency bands: delta (1–4 Hz), theta (4–7 Hz), alpha (8–13 Hz), beta (14–30 Hz), and gamma (30–45 Hz).

### Power spectrum analysis

Mean frequency (*MF*) and frontality laterality index (*LI*) were measured. The calculation formulas were defined as follows:


$$\begin{array}{l} \bar{f}=\frac{\overset{100}{\underset{f=1}{\sum }}\left(P\left(f\right)\times f\right)}{\overset{100}{\underset{f=1}{\sum }}P\left(f\right)} \end{array}$$

$$\begin{array}{l} LI=\frac{P_{\text{left}}-P_{\text{right}}}{P_{\text{left}}+P_{\text{right}}}. \end{array}$$

*P*_*left*_ is the average of the power spectrum of left frontal electrodes (Fp1, F3, and F7), while *P*_*right*_ is the average of the power spectrum of right frontal electrodes (Fp2, F4, and F8).

### EEG network analyzing pipeline

We adopted an analyzing pipeline (summarized in Supplementary Table S1) created in our lab to quantitate and source localized the EEG functional network, including a weighted classical network, *MST*, community structure, and sLORETA.

### Weighted classical network

#### Functional connectivity

Phase lag index, an index of asymmetry in the phase difference distribution calculated from the instantaneous phases of two-time series, was used to statistically quantify the phase synchronization of the two signals (34). The Hilbert transform was used to determine the instantaneous phase, and the Hann window was applied before performing the concurrent fast Fourier transform. When Δϕ is defined as the phase difference, *PLI* is calculated as follows:


PLI=|<(sign(Δϕ))>|

where the symbol “||” refers to taking the absolute value, the character “< >” means the average operator and the “sign” is the sign function in math.

#### Construction of brain network

We defined the EEG electrodes as network nodes, and the 1/*PLI* values between the paired electrodes were characterized as the edge distance. The functional connectivity matrix was calculated once for each epoch. All individual connectivity matrices were calculated and averaged to represent the network connections for the group to improve reliability.

The frontotemporal region network was constructed with Fp1, Fp2, F3, F4, F9, F10, T9, T10, P9, P10, F7, F8, T7, T8, P7, and P8 electrodes as nodes.

#### Small-world properties

After constructing the brain network, *CC* and characteristic *PL* were calculated (Supplementary Appendix S1). *CC* was a measure of the local segregation of the network. *PL* was an indicator of the overall integration, and a lower *PL* indicated a more integrated network.

### Minimum spanning tree

The minimum spanning tree is an acyclic subgraph connecting all the nodes in the weighted network, which extracts the network's backbone and reduces the influence of noise. Here, 1/*PLI* is considered the edge distance; then, the Kruskal algorithm is used to generate the MST. Five commonly used parameters were calculated in this study.

#### Diameter

The diameter (*D*) is measured as the distance between any two nodes of the tree.

#### Leaf fraction

The leaf fraction (*LF*) is the fraction of nodes with degree = 1 in *MST*.

#### Betweenness centrality

Betweenness centrality (*BC*) is the fraction of all paths on a tree that passes through a particular node. A node with a large betweenness is considered to play a crucial role in the network. The formula for calculating *BC* is as follows:


$$\begin{array}{l} BC\left(v_{i}\right)=\underset{s\neq t\neq v_{i}}{\sum }\frac{\sigma_{st}\left(v_{i}\right)}{\sigma_{st}} \end{array}$$

where σ_st_ is defined as the number of shortest paths from node s to node t, of which passing through V_i_ is expressed as σ_st_ (V_i_).

#### Tree hierarchy

The tree hierarchy (*TH*), which is characterized as a hierarchical metric that quantifies the trade-off between large-scale integration, is defined as follows:


$$\begin{array}{l} TH=\frac{LN}{2mBCmax} \end{array}$$

where *LN* is the leaf number of MST *and BC*_*max*_ is the largest betweenness centrality in the network.

#### Kappa

Kappa is a measure of the broadness of the degree distribution.

### Community structure

A community refers to dividing the nodes in the network into several relatively independent modules. The group connections were relatively dense, and the connections between the groups were relatively sparse (35). Modularity (*Q*) was used to measure the quality of the community partition. The larger the *Q*, the clearer the community division structure behaves. The calculation formula for *Q* is as follows:


$$\begin{array}{l} Q=\frac{1}{2M}\underset{i}{\sum }\underset{j}{\sum }\left(a_{ij}-\frac{k_{i}k_{j}}{2M}\right)\delta \left(C_{i}{,C}_{j}\right) \end{array}$$

where *M* represents the number of nodes and *M* represents the number of channels. *a*_*ij*_ represents the adjacency matrix and *k*_*i*_ represents the degree of node *i*. *Ci* represents the community assignment. *δ(Ci, Cj)* characterizes the different community assignments if nodes *i* and *j* belong to the same community, then *δ(Ci, Cj)* equals 0. In contrast, *δ(Ci, Cj)* equals 1 when *i* and *j* belong to different communities.

Partition coefficient (*PC*) measures the node centrality within and between the modules, where the large *PC* means the node plays essential roles in the community—the *PC* node *i* is defined as follows (36).


$$P_{i}=1-{\sum_{s=1}^{N_{M}}\left(\frac{\kappa_{is}}{k_{i}}\right)}^{2}$$

where *k*_*is*_ is the number of nodes *i* in module *s* and *k*_*i*_ is the degree of node *i*. *P*_*i*_ is closer to 1 if the links are uniformly distributed among modules. It is 0 when the node is connected only in their module.

### EEG source localization

sLORETA software (version. 20190617, https://www.uzh.ch/keyinst/loreta) was used to reconstruct the EEG signals. LORETA's algorithm is a linear inverse solution of the EEG signal. Under ideal conditions (no noise), there was no positioning error for the point source. The 3D distribution of the cortex was used to calculate the standardized current source density based on the potential distribution recorded on the scalp. Based on the Brodmann area (BA), differences in the functionally related brain areas were displayed on the Talairach template for three-dimensional display. We first created a list of 25 electrodes as EEG data were recorded, then made the transformation matrix, and computed the sLORETA images of the current density values for each voxel.

**Figure 1:**
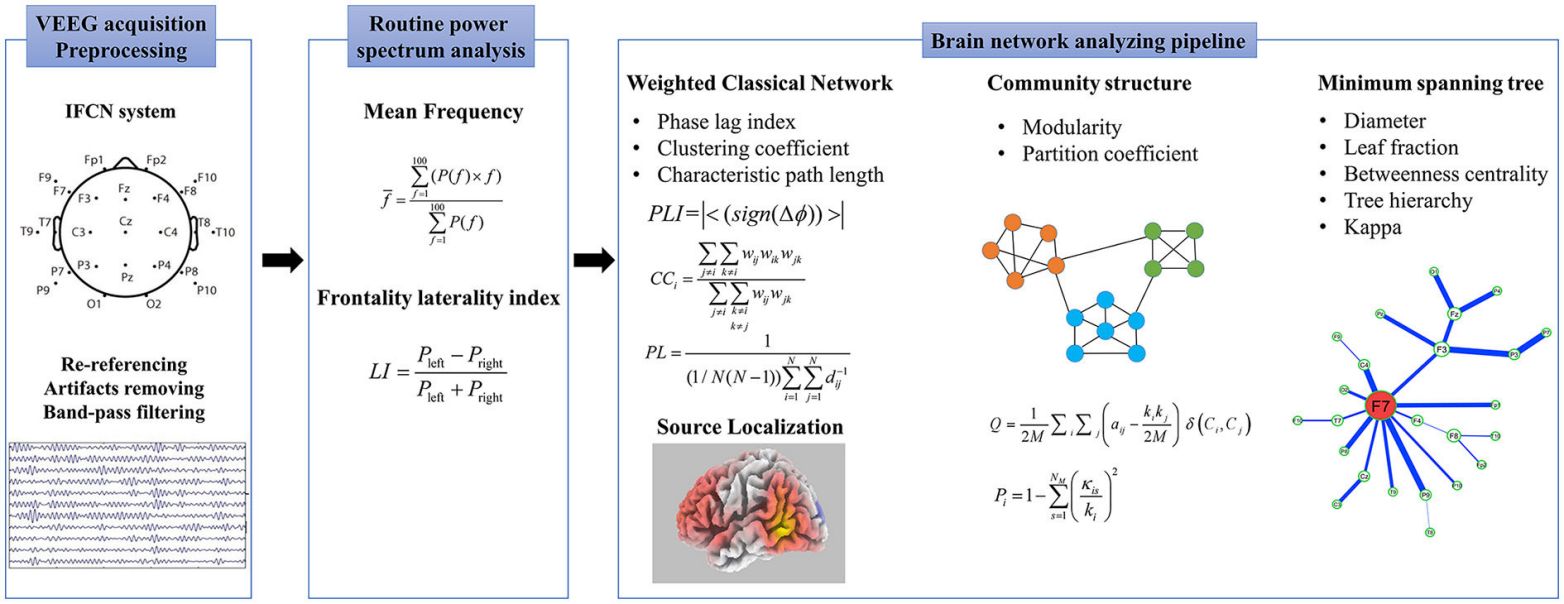
Protocol for electroencephalography processing and analyzing.

The procedure and all the parameters calculated are shown in Figure 1. The EEG analysis was blindly performed by the experienced engineering doctor, Gaoxing Zheng.

### Statistical methods

Numeric variables are expressed as mean ± standard deviation (SD) or median (interquartile range [IQR]). Statistical analysis was performed using SPSS software (version 25.0; SPSS Inc., Chicago, IL, USA). An ANOVA test with Tukey's *post-hoc* test was performed to differentiate demographics and EEG data among variable lateralization of patients with TLE and controls. Correlation analysis was performed using Spearman's correlation analysis. Multiple linear regression analysis was used to detect the attributing factors. The significance level for all tests was *p* < 0.05.

## Results

### Demographics and clinical data

A cohort of 151 patients with TLE (55 right, 56 left, and 40 bilateral) and 36 controls was enrolled. There were no significant differences in age (control: 38.44 ± 17.50, TLE: 41.47 ± 18.25, R-TLE: 41.87 ± 17.24, L-TLE: 39.05 ± 19.13, B-TLE: 44.30 ± 18.35) and gender (male/total, control: 17/36, TLE: 88/151, R-TLE: 33/55, L-TLE: 35/56, B-LTE: 20/40) between the groups (*P* > 0.05).

The age of onset of patients with TLE was 34.46±20.79 (30[32.25]) years old, and the disease duration was 7.17 ± 9.14 (4[9]) years. Sixty-five patients with TLE were treated with monotherapy, 25 were with two epileptic drugs (ASMs), 15 were with more than ASMs, and the other 46 patients did not receive any ASM. The ASM schedules were as follows: 38 patients were treated with valproic acid (VPA), 28 patients with carbamazepine (CBZ), eight patients with topiramate (TPM), 19 patients with levetiracetam (LEV), 20 patients with oxcarbazepine (OXC), four patients with phenobarbital (PB), 29 patients with lamotrigine (LTG), and one patient with phenytoin (PHT). Among them, 10.60% (16/151) had a history of febrile seizures, and 2.64% (4/151) had a family history of epilepsy.

Seizure severities were evaluated by seizure frequency, the diagnosis of DRE, and VA-2 scores. A total of 19 patients with TLE underwent a seizure frequency of more than 1 per week, 38 patients of no <1 per month, 67 patients of no more than 1 per year, and the remaining 27 patients had less than one seizure per year. In total, 33 patients were diagnosed with DRE according to the definition of ILAE 2010. The average of VA-2 score was 203.05 ± 378.91 (40[182]).

According to the ILAE 2017 classification criteria, 26 patients with TLE were classified as focal seizures, and the other 125 patients with TLE were assorted as focal to bilateral tonic–clonic seizures. A total of 104 patients were diagnosed with unknown etiology with negative routine MRI, and 47 with structural causes, of which 15 were HS, 1 was focal cortical dysplasia (FCD), 5 were encephalitis, 1 was trauma, 16 were occupying lesions, 3 were unknown temporal lobe atrophy, 4 were cerebrovascular diseases, and the other 2 were double pathology diagnosed by postoperative pathology: ganglioglioma (GG) with FCD type IIIb, and HS with cavernous hemangioma, respectively.

### Power spectrum analysis in patients with TLE

In routine power spectrum analysis, the interictal mean frequency demonstrated no significant differences existed among the TLE groups and controls (*F* = 0.791, *P* > 0.05). The *LI* results showed that only decreased frontal *LI* was found in L-TLE compared to patients with R-TLE (−0.002 ± 0.232 vs. 0.109 ± 0.160, *P* = *0.017*).

### Frontotemporal changes of resting network in patients with TLE

**Figure 2:**
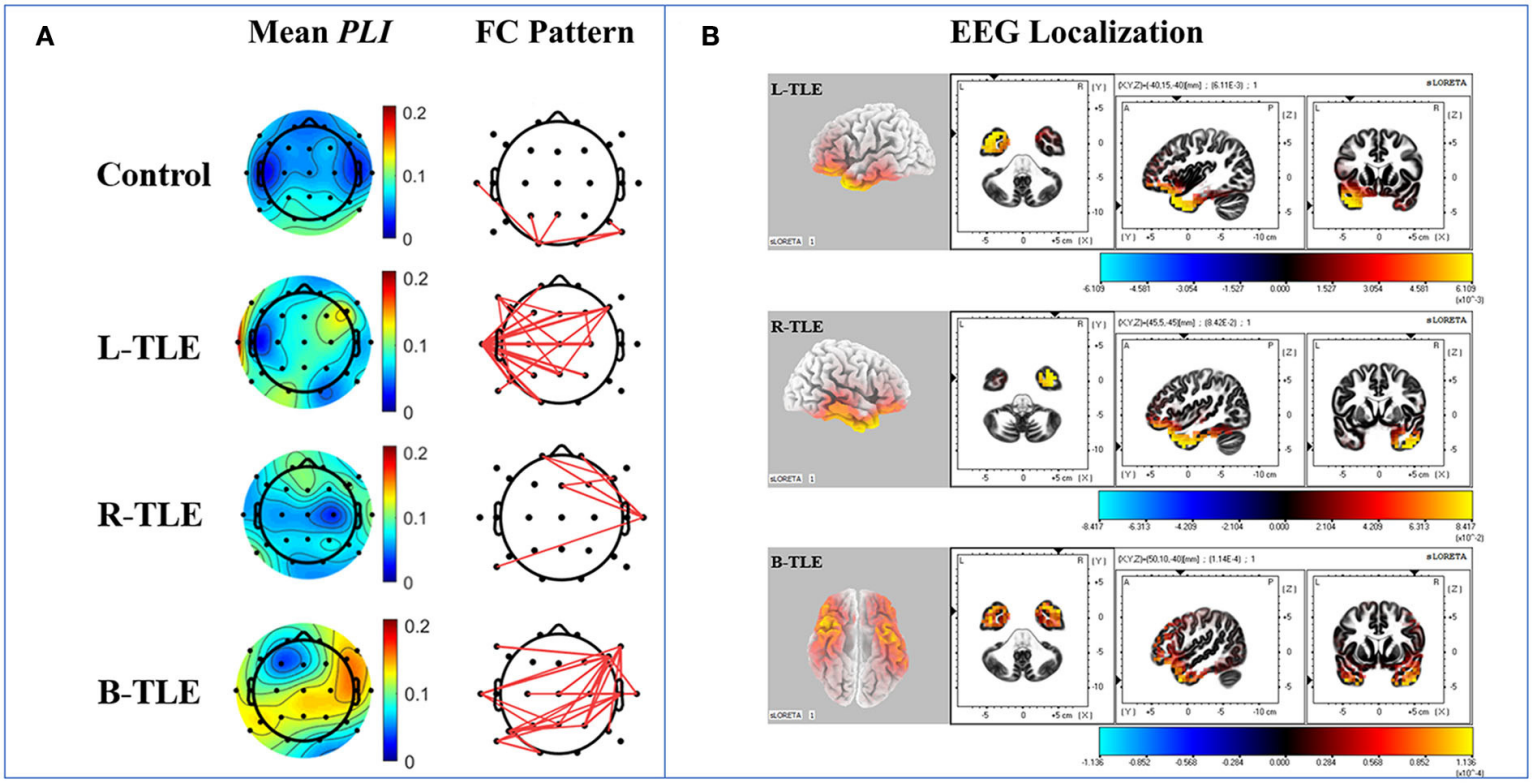
Diagrams of the functional network in patients with TLE and controls. The graph of the mean phase lag index, functional connection pattern **(A)** and sLORETA **(B)** displayed ipsilateral enhancement in frontotemporal regions in patients with TLE.

Using our network analysis pipeline, increased frontotemporal connectivity ipsilaterally was demonstrated in patients with TLE according to mean *PLI* and FC pattern diagrams, while the bilateral occipital increase was found in the controls (Figure 2A). Furthermore, the results of EEG source localization displayed ipsilateral frontotemporal enhancement in patients with TLE. The mainly involved brain regions of the patients with TLE were the superior (BA 38), middle (BA 21), and inferior (BA 20) temporal gyrus, inferior (BA 47, 45) and middle (BA 9, 10, 11) frontal gyrus (Figure 2B).

**Figure 3:**
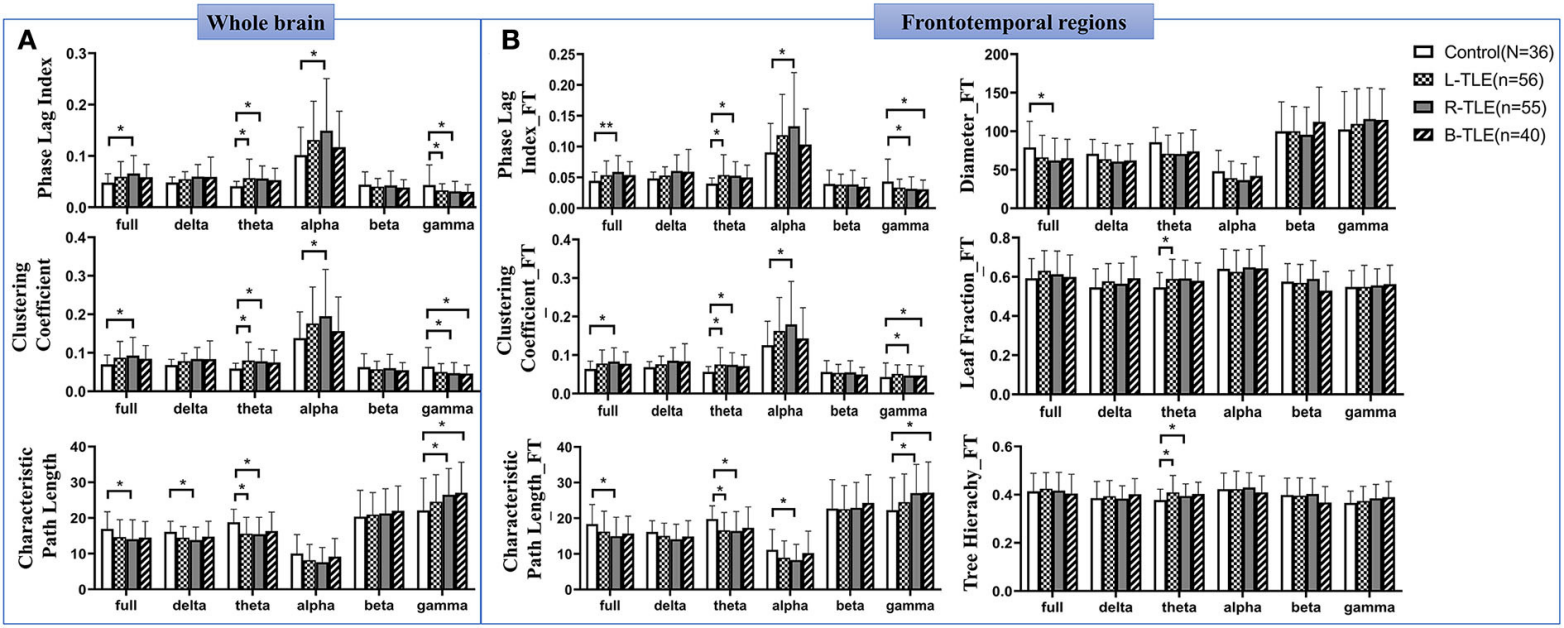
Alterations of the whole and frontotemporal network in patients with TLE. Differences of the classical weighted network in the whole brain among patients with TLE and the controls **(A)**. Alterations of the frontotemporal network in patients with TLE **(B)**. * *P* <0.05; ** *P* < 0.01.

Significantly changed global and frontotemporal weighted network values (increased *PLI, CC*, and decreased *PL*) were found in the theta band in patients with L-TLE and R-TLE, as well as in full and alpha bands in patients with R-TLE. In contrast, the network characteristics of patients with R-TLE show the opposite trend in the gamma band. (Figure 3, Supplementary Table S2).

Changed *MST* parameters were shown in frontotemporal regions. Reduced MST diameters were found in the full band. In the theta band, increased *TH* was found in L-TLE and R-TLE, and increased *LF* in L-TLE as well (Figure 3B, Supplementary Table S3).

### Interictal frontotemporal PLI values in the theta band are independently correlated with seizure severity

Increased frontotemporal *PLI* and *CC*, and decreased *PL* values in the theta band were observed in TLE patients with seizure frequency of ≥ 1 per month (*P* < 0.01), DRE (*P* < 0.01), antiseizure medications (*P* < 0.05), and VA-2 scores ≥ 30 (*P* < 0.01). A decreased MST diameter was also found in patients with a higher seizure frequency (*P* = 0.003) and DRE (*P* = 0.016) (Supplementary Table S4, Supplementary Figure S2). Correlation analysis showed that the VA-2 scores were positively correlated with the *PLI* (*r* = 0.254, *P* = 0.002) and *CC* (*r* = 0.242, *P* = 0.003) in the frontotemporal theta band and negatively correlated with *PL* (*r* = −0.248, *P* = 0.002) and diameter of *MST* (*r* = −0.187, *P* = 0.021).

We carried out a multivariate linear regression analysis to identify the most significant factors affecting seizure severity. We set VA-2 scores as a dependent variable, and functional network parameters in variable bands, patients' age, duration, age of onset, family history, history of febrile convulsions, seizure symptoms (including aura, seizure performance, post-seizure status, duration), ASM schedules (including numbers of ASM; VPA, CBZ, TPM, LEV, OXC, LTG, PHT, and PB usage), and history of status epilepticus as independent variables. The results showed that frontotemporal *PLI* values in the theta band (*β* = 0.259, *P* = 0.001) and age of onset (*β* = −0.215, *P* = 0.007) entered the regression model of VA-2 scores.

Considering the possible impact of lesions on brain networks, we further measured the multivariate analysis in 104 TLE patients with negative MRI and obtained consistent results: VA-2 scores were independently correlated with frontotemporal *PLI* values in the theta band (*β* = 0.263, *P* = 0.008) and age of onset (*β* = −0.195, *P* = 0.047). No significant difference was detected in frontotemporal PLI values in the theta band between the TLE patients with (0.559 ± 0.272) and without lesions (0.508 ± 0.280; *P* > 0.05). No difference was found between the patients with and without the certain ASM either (Supplementary Table S5).

## Discussion

Temporal lobe epilepsy is a common type of DRE that involves complex network mechanisms. Here, we adopted a graph theory analyzing pipeline based on EEG, including the weighted functional network, *MST*, and community structure analysis methods. We reported the independent correlations between frontotemporal functional connection and seizure severity in patients with TLE. This study highlighted the crucial role of the frontal lobe in TLE and provided a non-invasive and easy-to-use indicator for drug-resistant patients with TLE.

Compared to negative findings using routine power spectrum analysis, the graph theory network results showed increased frontotemporal connections ipsilaterally during interictal-resting periods. The frontal and temporal cortices are connected via various fibers, such as the AF and UF (14). Whether the frontal lobe participates in seizure propagation in patients with TLE remains unknown. Some studies have reported the involvement of the frontal lobes in patients with TLE, with controversial conclusions. Ipsilateral hyperperfusion in regions was observed in patients with mTLE using single-photon emission computed tomography. At the same time, significant hypoperfusion was found in the bilateral prefrontal areas (37). However, decreased frontal connectivity has also been detected during seizures (38). Other studies reported that frontotemporal, especially orbitofrontal, connections were interictally increased after interictal epileptic discharges (39). In this study, sLORETA images, according to EEG sources, a newer tomographic method to calculate electrical neuronal activity, were consistent with those connected by AF and UF. The pars triangularis (BA 45), posterior frontal gyrus (BA 8, 9), and pars opercularis (BA 44) were the regions in which the largest branch of AF was terminated (40). The other branch connects the posterior part of the superior temporal gyrus (BA 41, 42) to the inferior frontal gyrus (BA 44, 45) (41). The UF connects the anterior temporal lobe (BA 38) with the orbitofrontal (BA 11, 47) and frontal pole cortex (BA 10). The ventral branch of the UF terminates in the orbitofrontal cortex (BA 11, 47). In addition, the anterior and middle branches connect the anterior cingulate gyrus (BA 32) and frontal pole (BA 10) (41). These regions co-activated in R-TLE, L-TLE, and B-TLE may indicate the frontotemporal reconfigurations of the epileptic functional network. Because the presence of structural abnormality affects the epileptic network, the patients with abnormal extratemporal abnormalities on routine MR were excluded from this study to avoid confounding factors such as non-epileptogenic lesions. For the temporal lesions, multivariate analysis showed consistent results in 104 TLE patients with negative MRI.

Compared to other bands, the theta band was the most significant in the functional network in this study. Previous studies reported a unique theta oscillation in the human hippocampus, which was widely associated with attention, execution, and emotion (42). Little attention has been paid to epilepsy research. Theta rhythm was previously reported to regulate the firing frequency and discharge time of individual neurons (43). A study of 10 patients with DRE found that small-world parameters in the theta band were increased before seizures (44). Seizure behavior was related to theta activity in the kainic acid model (45). All the results indicate that the theta band plays an important role in seizure propagation.

The most important clinically relevant finding was that using our analyzing pipeline, frontotemporal *PLI* values in the theta band with IFCN-standard EEG were independently correlated with VA-2 scores. *PLI* values between 0 and 1 were used to describe brain synchronization and reported to be much better to avoid volume conduction than other parameters (synchronization likelihood, Fourier coherence coefficient, etc.) (46). Although weighted *PLI* (*wPLI*) is slightly better than *PLI* in avoiding volume conduction, Christodoulakis, et al. (47) addressed that the brain network indicators calculated based on *wPLI* are not significantly different from those calculated based on *PLI*. In addition, they emphasized that excessive pursuit of reducing the impact of volume conduction will result in a loss of the ability to identify abnormal epilepsy EEG changes. Therefore, we adopt the method of *PLI*, which can not only effectively avoid the impact of volume conduction, but also sensitively capture the network significance in patients with TLE compared to the controls. The larger the *PLI* was, the more vital synchronization was observed in the brain. Synchronized neurons are critical for brain function, whereas excessive synchronization is notably related to epilepsy (48). Meanwhile, increased *CC* and decreased *PL* were observed, indicating that local and global network connectivities were abnormal. The changed small-world topologies in patients with TLE were consistent with previous studies (19, 49), except for some controversial results (20, 50). These inconsistent results might be due to the different network types (structural or functional, global, or local network) or neuroimaging modalities (EEG or MRI). *MST* results showed that the frontotemporal diameter in patients with epilepsy was shorter than that in the controls. We observed that alterations in the frontotemporal *MST* parameters were more notable than those in the whole brain. However, in this study, we did not detect its sensitivity compared to the weighted-graph indicators, which might have been because of its focus on the network skeleton and ignorance of the weaker connections (51). In terms of prognosis prediction, other methods such as machine learning were preliminarily applied. Croce et al. (52) provide a pipeline using a machine learning approach for predicting the levetiracetam response in 23 patients with epilepsy. Tang et al. (53) develops and validates a machine learning-powered approach in neuroimaging for surgery outcome prediction. However, it remained to be improved in the clinical application, such as excessive feature extraction, computational cost, time consumption, the risk of overfitting in small datasets, etc. The changes in TLE frontotemporal network characteristics that we have discovered so far may guide us to use machine learning methods to predict clinical outcomes in the future.

According to the previous EEG brain network research, the epoch length of the EEG signal will affect the functional connectivity values, which in turn affects the brain network analysis results. The brain functional connectivity tends to be stable when the continuous EEG epoch is longer than 10 s (25, 54). Consistent with our previous study, 90-s EEG epochs were segmented and analyzed (55). At present, the latest electrophysiology network modeling analysis mainly depends on invasive EEG monitoring or high-density EEG (56). However, these were limited by routine clinical use and individualized implantation schemes. In 2017, the IFCN recommended a new electrode positioning system for routine practice. It was a 25-channel system, adding an inferior temporal chain based on the conventional 10–20 system (33). It was reported to have the same effect as high-density recordings (above 128 channels) in diagnostic yield (57). It could compensate for the lack of routine practice and individualization compared to SEEG and high-density EEG. We followed the IFCN guidelines to identify alterations within the temporal and frontal lobes. This study provided a non-invasive indicator with IFCN-system EEG for seizure severity, which was more accessible and standardized in routine practice.

The limitation of the present study is the insufficient analysis of the EEG network to describe the anatomical spatial characteristics. A better network characteristic could be described with a high-density EEG recording. Further work is required for the comparison of our data with SEEG and high-density EEG results. Second, it is a lack of accuracy for the source-level analysis using standard coordinates of electrodes. We will obtain the source location coordinates from each individual MRI and then transform them in Talairach space to precisely determine the distribution of region of source involvement in the following studies. Moreover, the predicted model will be acquired for multi-dimensional parameters, large sample sizes, and multi-center research.

In summary, our network analysis illustrated that ipsilateral frontotemporal regions are activated in the interictal state in patients with TLE. The frontotemporal *PLI* values in the theta band independently correlated with VA scores in patients with TLE. We suggested that increased frontotemporal theta synchronicity might be correlated with seizure severity. This study introduced a non-invasive method with routine scalp EEG for seizure severity and guided the treatment strategy for drug-resistant epilepsy.

## Data availability statement

The raw data supporting the conclusions of this article will be made available by the authors, without undue reservation.

## Ethics statement

The studies involving human participants were reviewed and approved by Zhongshan Hospital, Fudan University. The patients/participants provided their written informed consent to participate in this study.

## Author contributions

LM: study concept and design, acquisition of data, study coordination, and drafting. GZ: data analysis, interpretation, and revising the article. YC: data interpretation. WL: acquisition of data. QZ: acquisition of data. WP: acquisition of data. JD: study design, interpretation of data, and revising the article for content. XW: study concept and design and revising the article for content. All authors contributed to the article and approved the submitted version.

## Funding

This work was supported by Project grants from the Shanghai Municipal Committee of Science and Technology (Codes 17411962500, 16JC1420201, and 2018BR05).

## Conflict of interest

The authors declare that the research was conducted in the absence of any commercial or financial relationships that could be construed as a potential conflict of interest.

## Publisher's note

All claims expressed in this article are solely those of the authors and do not necessarily represent those of their affiliated organizations, or those of the publisher, the editors and the reviewers. Any product that may be evaluated in this article, or claim that may be made by its manufacturer, is not guaranteed or endorsed by the publisher.

## Abbreviations

ASM: antiseizure medication
AF: arcuate fasciculus
BA: Brodmann area
BC: betweenness centrality
CBZ: carbamazepine
CC: clustering coefficient
D: diameter
FC: functional connectivity
FIR: finite impulse response
ICA: independent component analysis
IEDs: interictal epileptic discharges
LF: leaf fraction
LEV: levetiracetam
LTG: lamotrigine
MDR: multidrug resistance
MST: minimum spanning tree
OXC: oxcarbazepine
PB: phenobarbital
PC: partition coefficient
PL: characteristic path length
PLI: phase lag index
Q: modularity
SEEG: stereoelectroencephalography
sLORETA: standardized low-resolution brain electromagnetic tomography analysis
TH: tree hierarchy
TLE: temporal lobe epilepsy
TPM: topiramate
UF: uncinate fasciculus
VA-2: veterans administration rating scale for seizure type and frequency
VPA: valproic acid.
