# Supplementary material for: Frontotemporal phase lag index correlates with seizure severity in patients with temporal lobe epilepsy
Source: Front Neurol. 2022 Dec 1;13:855842. doi: 10.3389/fneur.2022.855842 (PMC9752927; doi:10.3389/fneur.2022.855842)
Supplement: Supplementary file 1 [file Table_1.DOCX]

Supplementary Material

# Supplementary Figures and Tables

## Supplementary Figures

**Supplementary Figure S1** The standard electrode position according to conventional 10-10 system and IFCN system.

**Supplementary Figure S2.** Frontotemporal network in theta band between different groups of seizure severity.

## Supplementary Tables

**Supplementary Table S1.** Network metrics summary.

**Supplementary Table S2.** Changes of whole-brain and frontotemporal weighed network in patients with temporal lobe epilepsy

**Supplementary Table S3.** Alterations of minimum spanning tree and community structure in whole brain and frontotemporal regions in TLE patients

**Supplementary Table S4.** Frontotemporal network in theta band between different groups of seizure severity

**Supplementary Table S5.** Frontotemporal PLI values in theta band between different groups of the certain ASM

# Supplementary Appendix

**Supplementary Appendix 1.** The formulas for calculating *CC* and *PL*


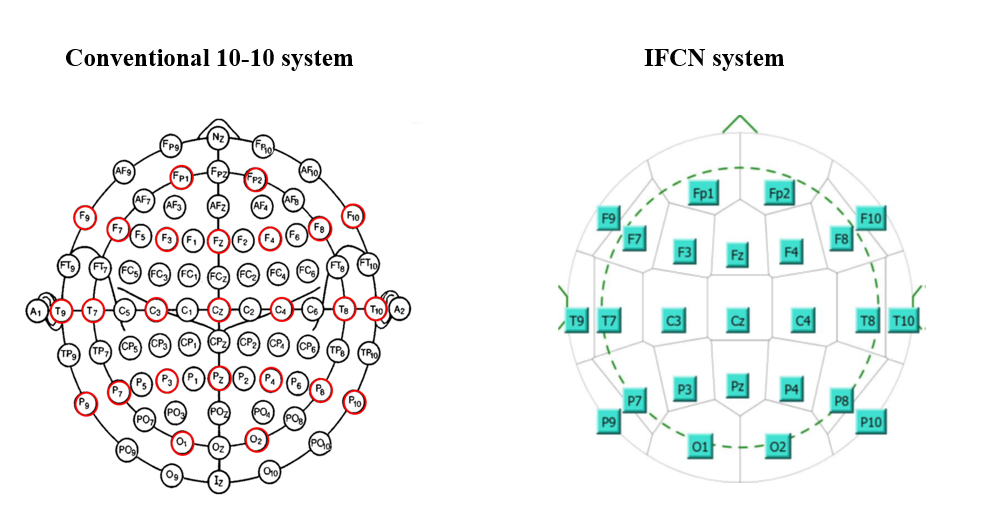


**Supplementary Figure S1.** **The standard electrode position according to conventional 10-10 system and IFCN system.**

The red circles represent the electrode placement positions according to the IFCN standard in this study displayed on the 10-10 atlas.


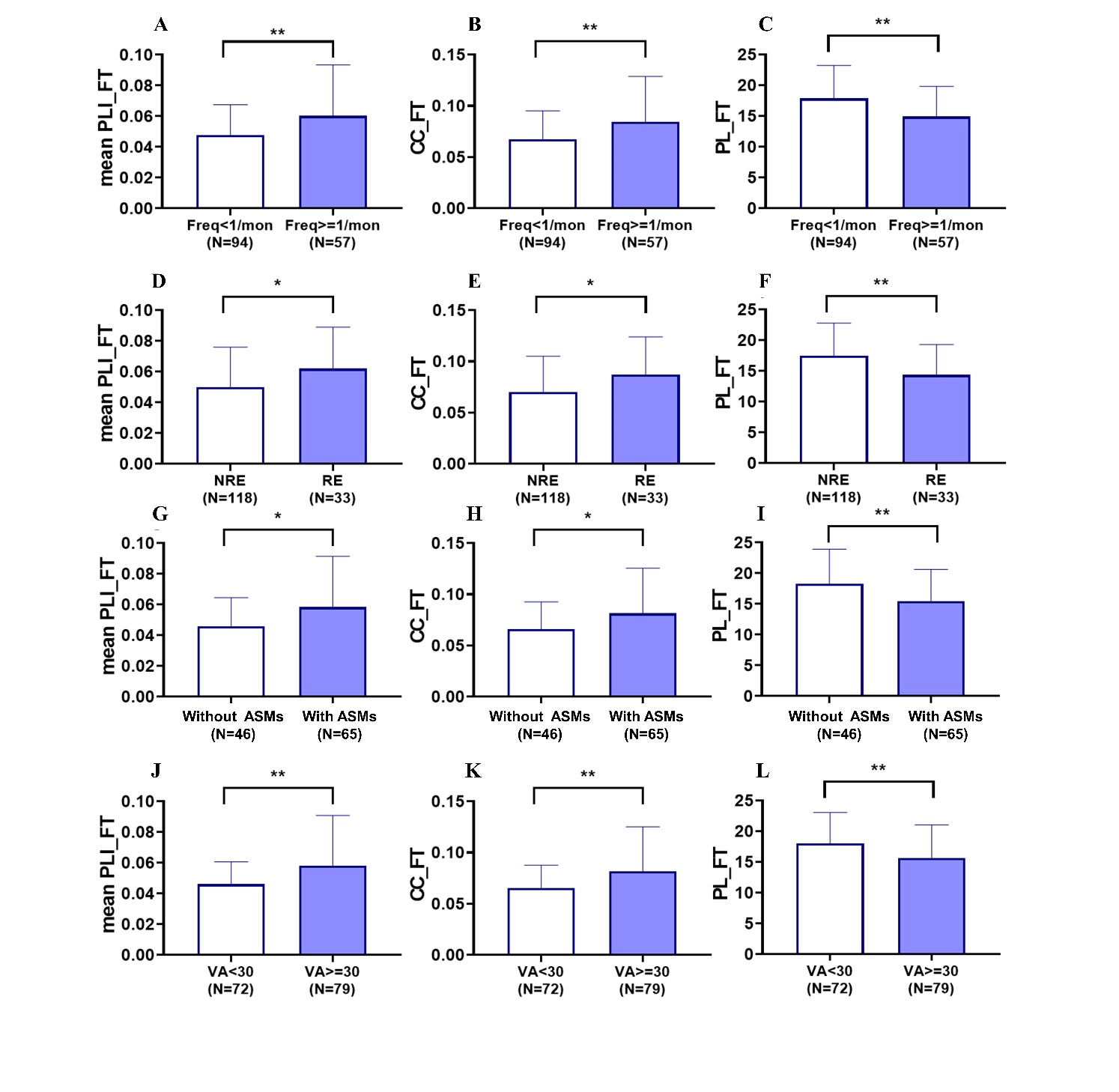


**Supplementary Figure S2.** **Frontotemporal network in theta band between different groups of seizure severity.**

Increased frontotemporal PLI and CC and decrease PL values were higher in TLE patients with seizure frequency ≥ 1 per month (**A-C**), with drug-resistant epilepsy (**D-F**), with antiseizure medications (**G-I**), and with VA scores ≥ 30 (**J-L**), compared to those with frequency < 1 per month, without RE, without use of antiseizure medications, and with VA scores-2 < 30 respectively; ** *P <* 0.01；* *P <* 0.05

**Supplementary Table S1 Network metrics summary.**

| Graph type | Matrix | | Definition |
| --- | --- | --- | --- |
| Weighted graph | *PLI* | Phase lag index | An index of asymmetry in the phase difference distribution is calculated from the instantaneous phases of two-time series. |
|  | *CC* | Clustering Coefficient | The total number of edges that are connected to the neighboring nodes. |
|  | *PL* | Path Length | The minimum number of edges connecting any two nodes in the network. |
|  |  |  |  |
| Minimum spanning tree | *D* | Diameter | The largest distance between any two nodes of the tree. |
|  | *LF* | Leaf Fraction | The fraction of nodes with degree = 1. |
|  | *TH* | Tree Hierarchy | A hierarchical metric quantifies the trade-off between large scale integration. |
|  | Kappa | | The broadness of the degree distribution. |
|  | *BC* | Betweenness Centrality | The fraction of all paths on the tree that pass through a particular node. |
|  |  |  |  |
| Community structure | *Q* | Modularity | An indication to measure the quality of community division proposed by Newman |
|  | *Pi* | Participation Coefficient | The degree of uniformity of node connection in all communities |

**Supplementary Table S2 Changes of whole-brain and frontotemporal weighed network in patients with temporal lobe epilepsy**

|  |  | **Whole brain** | | | | **Frontotemporal regions** | | | |
| --- | --- | --- | --- | --- | --- | --- | --- | --- | --- |
|  |  | Control | L-TLE | R-TLE | B-TLE | Control | L-TLE | R-TLE | B-TLE |
| PLI | full | 0.048 ± 0.017 | 0.060 ± 0.030  *P* > 0.05 | 0.066 ± 0.035  ***P =* 0.012** | 0.059 ± 0.025  *P* > 0.05 | 0.044 ± 0.014 | 0.054 ± 0.023  *P* > 0.05 | 0.059 ± 0.026  ***P =* 0.008** | 0.054 ± 0.022  *P* > 0.05 |
|  | delta | 0.048 ± 0.011 | 0.055 ± 0.014  *P* > 0.05 | 0.060 ± 0.023  *P* > 0.05 | 0.059 ± 0.039  *P* > 0.05 | 0.049 ± 0.010 | 0.053 ± 0.014  *P* > 0.05 | 0.061 ± 0.026  *P > 0.05* | 0.059 ± 0.036  *P* > 0.05 |
|  | theta | 0.041 ± 0.009 | 0.057 ± 0.037  ***P =* 0.019** | 0.056 ± 0.025  ***P =* 0.028** | 0.053 ± 0.023  *P* > 0.05 | 0.040 ± 0.009 | 0.054 ± 0.033  ***P =* 0.020** | 0.053 ± 0.023  ***P =* 0.033** | 0.050 ± 0.020  *P* > 0.05 |
|  | alpha | 0.102 ± 0.054 | 0.131 ± 0.076  *P* > 0.05 | 0.149 ± 0.102  ***P =* 0.031** | 0.117 ± 0.070  *P* > 0.05 | 0.091 ± 0.047 | 0.118 ± 0.066  *P* > 0.05 | 0.133 ± 0.087  ***P =* 0.012** | 0.103 ± 0.058  *P* > 0.05 |
|  | beta | 0.044 ± 0.025 | 0.040 ± 0.016  *P* > 0.05 | 0.043 ± 0.028  *P* > 0.05 | 0.038 ± 0.015  *P* > 0.05 | 0.040 ± 0.022 | 0.038 ± 0.017  *P* > 0.05 | 0.039 ± 0.023  *P* > 0.05 | 0.035 ± 0.014  *P* > 0.05 |
|  | gamma | 0.044 ± 0.039 | 0.033 ± 0.013  *P* > 0.05 | 0.031 ± 0.020  ***P =* 0.024** | 0.030 ± 0.014  ***P =* 0.028** | 0.043 ± 0.036 | 0.033 ± 0.014  *P* > 0.05 | 0.031 ± 0.019  ***P =* 0.030** | 0.031 ± 0.015  ***P =* 0.032** |
| CC | full | 0.070 ± 0.024 | 0.087 ± 0.042  *P* > 0.05 | 0.093 ± 0.048  ***P =* 0.022** | 0.085 ± 0.034  *P* > 0.05 | 0.064 ± 0.020 | 0.078 ± 0.035  *P* > 0.05 | 0.083 ± 0.036  ***P =* 0.013** | 0.078 ± 0.031  *P* > 0.05 |
|  | delta | 0.068 ± 0.014 | 0.078 ± 0.020  *P* > 0.05 | 0.084 ± 0.030  *P* > 0.05 | 0.084 ± 0.047  *P* > 0.05 | 0.068 ± 0.014 | 0.076 ± 0.021  *P* > 0.05 | 0.085 ± 0.034  *P* > 0.05 | 0.084 ± 0.046  *P* > 0.05 |
|  | theta | 0.059 ± 0.014 | 0.080 ± 0.048  ***P =*0.017** | 0.078 ± 0.032  ***P =* 0.038** | 0.075 ± 0.032  *P* > 0.05 | 0.056 ± 0.014 | 0.075 ± 0.044  ***P =* 0.019** | 0.074 ± 0.031  ***P =* 0.028** | 0.071 ± 0.029  *P* > 0.05 |
|  | alpha | 0.138 ± 0.068 | 0.176 ± 0.095  *P* > 0.05 | 0.195 ± 0.121  ***P =* 0.036** | 0.157 ± 0.088  *P* > 0.05 | 0.126 ± 0.062 | 0.162 ± 0.087  *P* > 0.05 | 0.180 ± 0.111  ***P =* 0.014** | 0.143 ± 0.080  *P* > 0.05 |
|  | beta | 0.063 ± 0.035 | 0.057 ± 0.021  *P* > 0.05 | 0.060 ± 0.036  *P > 0.05* | 0.054 ± 0.020  *P* > 0.05 | 0.056 ± 0.029 | 0.054 ± 0.022  *P* > 0.05 | 0.055 ± 0.030  *P* > 0.05 | 0.050 ± 0.019  *P* > 0.05 |
|  | gamma | 0.064 ± 0.049 | 0.050 ± 0.022  *P* > 0.05 | 0.047 ± 0.027  ***P =* 0.026** | 0.046 ± 0.022  ***P =*0.024** | 0.043 ± 0.036 | 0.051 ± 0.023  *P* > 0.05 | 0.047 ± 0.028  ***P =* 0.029** | 0.047 ± 0.025  ***P =* 0.043** |

**Supplementary Table S2 Changes of whole-brain and frontotemporal weighed network in patients with temporal lobe epilepsy(continued)**

|  | |  | **Whole brain** | | | | **Frontotemporal regions** | | | |
| --- | --- | --- | --- | --- | --- | --- | --- | --- | --- | --- |
|  | |  | Control | L-TLE | R-TLE | B-TLE | Control | L-TLE | R-TLE | B-TLE |
| PL | | full | 16.91 ± 4.83 | 14.60 ± 4.91  *P >* 0.05 | 14.05 ± 5.43  ***P =* 0.021** | 14.52 ± 4.50  *P >* 0.05 | 18.39 ± 5.45 | 16.26 ± 5.71  *P* > 0.05 | 14.98 ± 5.31  ***P =* 0.010** | 15.72 ± 4.87  *P* > 0.05 |
|  |  | delta | 16.10 ± 2.97 | 14.45 ± 3.18  *P >* 0.05 | 13.83 ± 3.63  ***P =* 0.017** | 14.76 ± 4.32  *P >* 0.05 | 16.16 ± 3.15 | 15.06 ± 3.54  *P* > 0.05 | 14.11 ± 4.20  *P* > 0.05 | 14.90 ± 4.43  *P* > 0.05 |
|  |  | theta | 18.82 ± 3.63 | 15.57 ± 4.61  ***P =* 0.003** | 15.47 ± 4.70  ***P =* 0.003** | 16.33 ± 5.30  *P >* 0.05 | 19.77 ± 3.72 | 16.62 ± 4.99  ***P =* 0.012** | 16.46 ± 5.42  ***P =* 0.008** | 17.31 ± 5.87  *P* > 0.05 |
|  |  | alpha | 10.03 ± 5.31 | 8.19 ± 4.39  *P* > 0.05 | 7.58 ± 4.13  *P* > 0.05 | 9.16 ± 5.08  *P >* 0.05 | 11.14 ± 5.75 | 8.93 ± 4.71  *P >* 0.05 | 8.28 ± 4.41  ***P =* 0.028** | 10.23 ± 6.14  *P* > 0.05 |
|  |  | beta | 20.36 ± 7.40 | 20.96 ± 6.17  *P* > 0.05 | 21.21 ± 6.97  *P* > 0.05 | 22.03 ± 6.93  *P >* 0.05 | 22.72 ± 8.11 | 22.56 ± 6.62  *P >* 0.05 | 22.90 ± 7.14  *P* > 0.05 | 24.32 ± 7.88  *P >* 0.05 |
|  |  | gamma | 22.11 ± 9.03 | 24.56 ± 7.56  *P >* 0.05 | 26.47 ± 7.38  ***P =* 0.031** | 27.05 ± 8.57  ***P =* 0.021** | 22.27 ± 9.13 | 24.52 ± 7.89  *P >* 0.05 | 27.03 ± 8.08  ***P =* 0.022** | 27.20 ± 8.57  ***P =* 0.028** |
| CC / CCr | | full | 1.043 ± 0.029 | 1.051 ± 0.034 | 1.038 ± 0.019 | 1.040 ± 0.030 | 1.041 ± 0.031 | 1.053 ± 0.036 | 1.048 ± 0.031 | 1.046 ± 0.039 |
|  |  | delta | 1.028 ± 0.016 | 1.040 ± 0.021 | 1.032 ± 0.021 | 1.038 ± 0.023 | 1.030 ± 0.027 | 1.043 ± 0.033 | 1.041 ± 0.039 | 1.038 ± 0.037 |
|  |  | theta | 1.031 ± 0.035 | 1.036 ± 0.022 | 1.028 ± 0.017 | 1.033 ± 0.021 | 1.025 ± 0.033 | 1.040 ± 0.029 | 1.040 ± 0.034 | 1.042 ± 0.037 |
|  |  | alpha | 1.047 ± 0.028 | 1.045 ± 0.025 | 1.040 ± 0.016 | 1.042 ± 0.022 | 1.053 ± 0.036 | 1.050 ± 0.030 | 1.047 ± 0.026 | 1.042 ± 0.023 |
|  |  | beta | 1.037 ± 0.024 | 1.043 ± 0.034 | 1.032 ± 0.021 | 1.033 ± 0.025 | 1.037 ± 0.037 | 1.041 ± 0.045 | 1.039 ± 0.047 | 1.029 ± 0.029 |
|  |  | gamma | 1.050 ± 0.046 | 1.044 ± 0.049 | 1.052 ± 0.055 | 1.046 ± 0.037 | 1.045 ± 0.038 | 1.043 ± 0.073 | 1.042 ± 0.057 | 1.035 ± 0.053 |
| PL / PLr | full | | 1.017 ± 0.021 | 1.024 ± 0.028 | 1.025 ± 0.027 | 1.029 ± 0.026 | 1.011 ± 0.026 | 1.020 ± 0.030 | 1.014 ± 0.029 | 1.022 ± 0.023 |
|  | delta | | 1.015 ± 0.016 | 1.015 ± 0.023 | 1.019 ± 0.018 | 1.012 ± 0.023 | 1.012 ± 0.021 | 1.014 ± 0.027 | 1.010 ± 0.023 | 1.014 ± 0.029 |
|  | theta | | 1.018 ± 0.027 | 1.020 ± 0.025 | 1.023 ± 0.017 | 1.022 ± 0.024 | 1.016 ± 0.022 | 1.017 ± 0.026 | 1.016 ± 0.024 | 1.014 ± 0.022 |
|  | alpha | | 1.025 ± 0.027 | 1.030 ± 0.026 | 1.026 ± 0.022 | 1.026 ± 0.022 | 1.023 ± 0.032 | 1.027 ± 0.030 | 1.025 ± 0.026 | 1.032 ± 0.027 |
|  | beta | | 1.023 ± 0.022 | 1.021 ± 0.026 | 1.023 ± 0.021 | 1.023 ± 0.022 | 1.024 ± 0.021 | 1.020 ± 0.030 | 1.024 ± 0.028 | 1.023 ± 0.022 |
|  | gamma | | 1.016 ± 0.032 | 1.014 ± 0.023 | 1.006 ± 0.025 | 1.009 ± 0.027 | 1.015 ± 0.032 | 1.013 ± 0.032 | 1.008 ± 0.034 | 1.013 ± 0.040 |

PLI: phase lag index; CC: clustering coefficient; PL: characteristic path length.

*P* value showed in this table was characterized as the difference compared to the control group. There was no statistical difference between different sides of TLE patients (*P >* 0.05), which was not listed in the table. There was no difference in CC / CCr and PL / PLr among the groups (*P >* 0.05)

**Supplementary Table S3 Alterations of minimum spanning tree and community structure in whole brain and frontotemporal regions in TLE patients**

|  |  | **Whole brain** | | | | **Frontotemporal regions** | | | |
| --- | --- | --- | --- | --- | --- | --- | --- | --- | --- |
|  |  | Control | L-TLE | R-TLE | B-TLE | Control | L-TLE | R-TLE | B-TLE |
| Diameter | full | 77.53 ± 26.56 | 67.95 ± 27.18 | 64.60 ± 25.94 | 66.70 ± 25.36 | 79.03 ± 33.87 | 66.14 ± 28.62 | **62.02 ± 25.85^a^** | 65.15 ± 24.35 |
|  | delta | 78.99 ± 17.62 | 68.43 ± 18.65 | 68.33 ± 23.03 | 69.86 ± 24.06 | 70.71 ± 18.72 | 63.51 ± 20.81 | 60.77 ± 20.92 | 62.15 ± 21.64 |
|  | theta | 88.72 ± 23.52 | 77.04 ± 28.24 | 76.40 ± 27.61 | 79.97 ± 33.13 | 85.78 ± 18.92 | 70.76 ± 24.20 | 70.50 ± 27.10 | 73.96 ± 27.82 |
|  | alpha | 47.46 ± 25.89 | 39.23 ± 22.05 | 37.06 ± 21.51 | 43.81 ± 24.93 | 48.04 ± 27.03 | 39.04 ± 22.00 | 36.53 ± 21.45 | 42.01 ± 24.81 |
|  | beta | 94.86 ± 36.83 | 101.47 ± 32.49 | 98.45 ± 38.72 | 107.38 ± 40.00 | 99.85 ± 38.80 | 99.97 ± 32.17 | 95.44 ± 35.79 | 112.21 ± 45.00 |
|  | gamma | 106.31 ± 50.83 | 116.82 ± 45.27 | 121.88 ± 40.38 | 126.40 ± 53.21 | 102.35 ± 49.16 | 109.42 ± 45.65 | 115.85 ± 40.50 | 114.60 ± 40.35 |
| Tree Hierachy | full | 0.424 ± 0.072 | 0.427 ± 0.053 | 0.423 ± 0.071 | 0.404 ± 0.062 | 0.414 ± 0.075 | 0.425 ± 0.067 | 0.417 ± 0.076 | 0.405 ± 0.080 |
|  | delta | 0.386 ± 0.060 | 0.397 ± 0.059 | 0.396 ± 0.057 | 0.402 ± 0.074 | 0.386 ± 0.069 | 0.394 ± 0.065 | 0.384 ± 0.053 | 0.402 ± 0.065 |
|  | theta | 0.385 ± 0.061 | 0.403 ± 0.057 | 0.394 ± 0.055 | 0.416 ± 0.061 | 0.378 ± 0.045 | **0.410 ± 0.070^b^** | **0.395 ± 0.050^c^** | 0.403 ± 0.049 |
|  | alpha | 0.440 ± 0.054 | 0.419 ± 0.063 | 0.428 ± 0.059 | 0.411 ± 0.055 | 0.423 ± 0.067 | 0.423 ± 0.075 | 0.430 ± 0.061 | 0.410 ± 0.068 |
|  | beta | 0.426 ± 0.054 | 0.407 ± 0.065 | 0.406 ± 0.053 | 0.415 ± 0.071 | 0.399 ± 0.070 | 0.396 ± 0.074 | 0.403 ± 0.065 | 0.368 ± 0.066 |
|  | gamma | 0.392 ± 0.055 | 0.388 ± 0.057 | 0.405 ± 0.054 | 0.400 ± 0.047 | 0.366 ± 0.049 | 0.374 ± 0.061 | 0.385 ± 0.058 | 0.390 ± 0.065 |
| Leaf Fraction | full | 0.616 ± 0.083 | 0.631 ± 0.085 | 0.612 ± 0.086 | 0.599 ± 0.095 | 0.593 ± 0.100 | 0.631 ± 0.103 | 0.613 ± 0.119 | 0.600 ± 0.112 |
|  | delta | 0.553 ± 0.067 | 0.580 ± 0.074 | 0.564 ± 0.082 | 0.584 ± 0.097 | 0.546 ± 0.095 | 0.577 ± 0.091 | 0.565 ± 0.105 | 0.593 ± 0.110 |
|  | theta | 0.567 ± 0.094 | 0.580 ± 0.083 | 0.572 ± 0.072 | 0.594 ± 0.078 | 0.546 ± 0.076 | **0.590 ± 0.100^d^** | 0.592 ± 0.093 | 0.580 ± 0.091 |
|  | alpha | 0.657 ± 0.071 | 0.642 ± 0.089 | 0.643 ± 0.073 | 0.632 ± 0.075 | 0.641 ± 0.101 | 0.626 ± 0.110 | 0.649 ± 0.092 | 0.643 ± 0.116 |
|  | beta | 0.602 ± 0.072 | 0.584 ± 0.084 | 0.593 ± 0.067 | 0.591 ± 0.082 | 0.576 ± 0.092 | 0.569 ± 0.095 | 0.590 ± 0.094 | 0.530 ± 0.097 |
|  | gamma | 0.578 ± 0.091 | 0.572 ± 0.093 | 0.593 ± 0.074 | 0.580 ± 0.078 | 0.548 ± 0.084 | 0.548 ± 0.111 | 0.556 ± 0.085 | 0.563 ± 0.097 |
| Kappa | full | 0.316 ± 0.050 | 0.304 ± 0.054 | 0.318 ± 0.049 | 0.319 ± 0.062 | 0.366 ± 0.051 | 0.344 ± 0.056 | 0.353 ± 0.054 | 0.357 ± 0.058 |
|  | delta | 0.351 ± 0.031 | 0.332 ± 0.049 | 0.344 ± 0.053 | 0.329 ± 0.059 | 0.389 ± 0.044 | 0.370 ± 0.045 | 0.371 ± 0.058 | 0.360 ± 0.061 |
|  | theta | 0.339 ± 0.059 | 0.340 ± 0.049 | 0.333 ± 0.046 | 0.334 ± 0.056 | 0.383 ± 0.043 | 0.366 ± 0.047 | 0.359 ± 0.051 | 0.378 ± 0.047 |
|  | alpha | 0.289 ± 0.050 | 0.289 ± 0.062 | 0.292 ± 0.047 | 0.290 ± 0.064 | 0.326 ± 0.058 | 0.342 ± 0.062 | 0.331 ± 0.052 | 0.328 ± 0.063 |
|  | beta | 0.326 ± 0.045 | 0.332 ± 0.049 | 0.333 ± 0.040 | 0.333 ± 0.048 | 0.368 ± 0.042 | 0.371 ± 0.047 | 0.365 ± 0.051 | 0.391 ± 0.041 |
|  | gamma | 0.328 ± 0.065 | 0.331 ± 0.062 | 0.323 ± 0.051 | 0.331 ± 0.048 | 0.371 ± 0.056 | 0.374 ± 0.057 | 0.380 ± 0.047 | 0.375 ± 0.050 |

**Supplementary Table S3 Alterations of minimum spanning tree and community structure in whole brain and frontotemporal regions in TLE patients(continued)**

|  |  | **Whole brain** | | | | **Frontotemporal regions** | | | |
| --- | --- | --- | --- | --- | --- | --- | --- | --- | --- |
|  |  | Control | L-TLE | R-TLE | B-TLE | Control | L-TLE | R-TLE | B-TLE |
| Betweenness Centrality | full | 0.205 ± 0.089 | 0.214 ± 0.095 | 0.192 ± 0.074 | 0.204 ± 0.099 | 0.237 ± 0.093 | 0.254 ± 0.109 | 0.255 ± 0.101 | 0.247 ± 0.112 |
|  | delta | 0.174 ± 0.060 | 0.195 ± 0.081 | 0.172 ± 0.087 | 0.192 ± 0.086 | 0.217 ± 0.074 | 0.233 ± 0.089 | 0.225 ± 0.103 | 0.249 ± 0.109 |
|  | theta | 0.189 ± 0.110 | 0.181 ± 0.072 | 0.161 ± 0.054 | 0.182 ± 0.076 | 0.221 ± 0.108 | 0.225 ± 0.091 | 0.233 ± 0.084 | 0.213 ± 0.096 |
|  | alpha | 0.208 ± 0.095 | 0.207 ± 0.096 | 0.198 ± 0.084 | 0.193 ± 0.090 | 0.240 ± 0.102 | 0.230 ± 0.112 | 0.227 ± 0.089 | 0.232 ± 0.089 |
|  | beta | 0.188 ± 0.072 | 0.200 ± 0.088 | 0.175 ± 0.060 | 0.185 ± 0.057 | 0.224 ± 0.081 | 0.237 ± 0.108 | 0.241 ± 0.102 | 0.220 ± 0.082 |
|  | gamma | 0.217 ± 0.122 | 0.237 ± 0.123 | 0.234 ± 0.121 | 0.224 ± 0.086 | 0.255 ± 0.113 | 0.266 ± 0.144 | 0.244 ± 0.113 | 0.254 ± 0.125 |
|  |  |  |  |  |  |  |  |  |  |
| Modularity | full | 0.199 ± 0.059 | 0.195 ± 0.060 | 0.196 ± 0.055 | 0.190 ± 0.052 | 0.205 ± 0.061 | 0.191 ± 0.053 | 0.197 ± 0.052 | 0.198 ± 0.069 |
|  | delta | 0.219 ± 0.037 | 0.225 ± 0.035 | 0.218 ± 0.043 | 0.216 ± 0.040 | 0.234 ± 0.046 | 0.238 ± 0.049 | 0.238 ± 0.058 | 0.225 ± 0.063 |
|  | theta | 0.235 ± 0.029 | 0.218 ± 0.042 | 0.222 ± 0.038 | 0.219 ± 0.042 | 0.268 ± 0.050 | 0.241 ± 0.054 | 0.243 ± 0.053 | 0.239 ± 0.057 |
|  | alpha | 0.185 ± 0.050 | 0.168 ± 0.045 | 0.168 ± 0.045 | 0.177 ± 0.055 | 0.193 ± 0.071 | 0.184 ± 0.056 | 0.182 ± 0.069 | 0.179 ± 0.072 |
|  | beta | 0.217 ± 0.046 | 0.213 ± 0.042 | 0.221 ± 0.043 | 0.222 ± 0.037 | 0.257 ± 0.056 | 0.242 ± 0.070 | 0.228 ± 0.059 | 0.259 ± 0.055 |
|  | gamma | 0.224 ± 0.046 | 0.225 ± 0.046 | 0.244 ± 0.043 | 0.231 ± 0.051 | 0.262 ± 0.059 | 0.268 ± 0.066 | 0.259 ± 0.054 | 0.269 ± 0.060 |
| Participation coefficient | full | 0.366 ± 0.067 | 0.365 ± 0.078 | 0.374 ± 0.068 | 0.373 ± 0.080 | 0.279 ± 0.075 | 0.301 ± 0.071 | 0.315 ± 0.067 | 0.320 ± 0.073 |
|  | delta | 0.411 ± 0.073 | 0.432 ± 0.061 | 0.399 ± 0.068 | 0.407 ± 0.069 | 0.359 ± 0.063 | 0.330 ± 0.076 | 0.340 ± 0.065 | 0.322 ± 0.074 |
|  | theta | 0.416 ± 0.085 | 0.404 ± 0.077 | 0.401 ± 0.069 | 0.438 ± 0.069 | 0.326 ± 0.065 | 0.320 ± 0.072 | 0.328 ± 0.070 | 0.322 ± 0.076 |
|  | alpha | 0.360 ± 0.080 | 0.372 ± 0.074 | 0.383 ± 0.069 | 0.388 ± 0.066 | 0.286 ± 0.083 | 0.306 ± 0.075 | 0.321 ± 0.066 | 0.307 ± 0.078 |
|  | beta | 0.401 ± 0.079 | 0.384 ± 0.076 | 0.400 ± 0.075 | 0.392 ± 0.071 | 0.305 ± 0.079 | 0.310 ± 0.075 | 0.305 ± 0.085 | 0.308 ± 0.089 |
|  | gamma | 0.429 ± 0.088 | 0.432 ± 0.071 | 0.414 ± 0.076 | 0.452 ± 0.072 | 0.302 ± 0.068 | 0.312 ± 0.081 | 0.329 ± 0.073 | 0.322 ± 0.080 |

a：*P* = 0.027 (compared with controls);

b：*P* = 0.025 (compared with controls);

c：*P* = 0.028 (compared with controls);

d：*P* = 0.037 (compared with controls)

**Supplementary Table S4 Frontotemporal network in theta band between different groups of seizure severity**

|  | PLI_FT | CC_FT | PL_FT | Diameter_FT |
| --- | --- | --- | --- | --- |
| Frequency ≥ 1 /month | 0.060 ± 0.033 | 0.085 ± 0.044 | 14.90 ± 4.91 | 63.59 ± 24.25 |
| Frequency <1 /month | 0.048 ± 0.020 | 0.067 ± 0.028 | 17.87 ± 5.35 | 76.31 ± 26.17 |
| *P value* | *P =* 0.004 | *P =* 0.004 | *P =* 0.001 | *P =* 0.003 |
|  |  |  |  |  |
| RE | 0.062 ± 0.027 | 0.087 ± 0.037 | 14.37 ± 4.90 | 61.67 ± 27.15 |
| NRE | 0.058 ± 0.033 | 0.070 ± 0.035 | 17.46 ± 5.31 | 74.48 ± 25.18 |
| *P value* | *P =* 0.015 | *P =* 0.017 | *P =* 0.003 | *P =* 0.016 |
|  |  |  |  |  |
| ASMs>0 | 0.058 ± 0.033 | 0.082 ± 0.044 | 15.49 ± 5.13 | 69.87 ± 25.60 |
| ASMs = 0 | 0.050 ± 0.026 | 0.066 ± 0.027 | 18.33 ± 5.59 | 75.25 ± 27.19 |
| *P value* | *P =* 0.023 | *P =* 0.033 | *P =* 0.007 | *P >* 0.05 |
|  |  |  |  |  |
| VA-2 ≥ 30 | 0.058 ± 0.033 | 0.082 ± 0.043 | 15.61 ± 5.41 | 68.50 ± 26.72 |
| VA-2 < 30 | 0.046 ± 0.015 | 0.066 ± 0.022 | 17.99 ± 5.07 | 74.82 ± 25.22 |
| *P value* | *P =* 0.003 | *P =* 0.005 | *P =* 0.006 | *P >* 0.05 |

**Supplementary Table S5 Frontotemporal *PLI* values in theta band between different groups of the certain ASM**

|  | PLI_FT | *P value* |
| --- | --- | --- |
| Without VPA | 0.050 ± 0.021 | 0.188 |
| With VPA | 0.059 ± 0.037 |  |
|  |  |  |
| Without CBZ | 0.050 ± 0.025 | 0.100 |
| With CBZ | 0.061 ± 0.032 |  |
|  |  |  |
| Without TPM | 0.053 ± 0.027 | 0.350 |
| With TPM | 0.044 ± 0.011 |  |
|  |  |  |
| Without LEV | 0.054 ± 0.028 | 0.101 |
| With LEV | 0.043 ± 0.011 |  |
|  |  |  |
| Without OXC | 0.052 ± 0.028 | 0.937 |
| With OXC | 0.052 ± 0.015 |  |
|  |  |  |
| Without LTG | 0.053 ± 0.028 | 0.613 |
| With LTG | 0.050 ± 0.015 |  |
|  |  |  |
| Without PB | 0.053 ± 0.027 | 0.499 |
| With PB | 0.044 ± 0.005 |  |

VPA = valproic acid; CBZ = carbamazepine; TPM = topiramate; LEV = levetiracetam; OXC = oxcarbazepine; PB = phenobarbital; LTG = lamotrigine

**Supplementary Appendix 1** The formulas for calculating *CC* and *PL*

## 1.1 The formula for calculating *CC* is as follows:

$$CC_{i}=\frac{\sum_{j\neq i} \sum_{k\neq i} w_{ij}w_{ik}w_{jk}}{\sum_{j\neq i} \sum_{\begin{matrix} k\neq i \\ k\neq j \end{matrix}} w_{ij}w_{jk}}$$ 

where w_ij_ is the weight (*PLI* value) of the edge between nodes i and j,

The *CC* of the entire network is the average of all nodes (N is the total number of electrodes).

$$CC=\frac{1}{N}\sum_{i=1}^{N} C_{i}$$ 

Normalized *CC* is calculated as *CC/CCrandom* (*CC/CCr*), where the *CC_random_* is calculated as the average clustering coefficient of 100 permutated networks.

## 1.2 PL is calculated as follows:

$$PL=\frac{1}{(1/N(N-1))\sum_{i=1}^{N} \sum_{j=1}^{N} d_{ij}^{-1}}$$ 

where *d_ij_* is the shortest path between nodes i and j, which can be obtained using Dijkstra’s algorithm(Skiena, 1990).

Normalized *PL* is defined as *PL/PLrandom* (*PL/PLr*), where *PL_random_* is obtained by the average *PL* of 100 permutated networks.

# Reference

Skiena, S. (1990). Dijkstra's algorithm. *Implementing Discrete Mathematics: Combinatorics and Graph Theory with Mathematica***,** 225-227.
